# Supplementary material for: Grave-to-cradle photothermal upcycling of waste polyesters over spent LiCoO2
Source: Nat Commun. 2024 Mar 28;15:2730. doi: 10.1038/s41467-024-47024-x (PMC10979025; doi:10.1038/s41467-024-47024-x)
Supplement: Supplementary file 1 — Supplementary Information [file 41467_2024_47024_MOESM1_ESM.pdf]

Supplementary Information

Grave-to-Cradle Photothermal Upcycling of Waste Polyesters over Spent $LiCoO_2$

Xiangxi Lou,[1,2,#] Penglei Yan,[1,#] Binglei Jiao,[3,#] Qingye Li,[1] Panpan Xu,[3,] Lei Wang,[1] Liang Zhang,[1] Muhan Cao,[1] Guiling Wang,[2] Zheng Chen,[4] Qiao Zhang,[1] Jinxing Chen[1,*]*

[1] Institute of Functional Nano & Soft Materials (FUNSOM), Jiangsu Key Laboratory for Carbon-Based Functional Materials & Devices, Soochow University, Suzhou, 215123, Jiangsu, China

[2] Key Laboratory of Superlight Materials and Surface Technology of Ministry of Education, College of Materials Science and Chemical Engineering, Harbin Engineering University, Harbin 150001, China

[3] Advanced Materials Division, Suzhou Institute of Nano-Tech and Nano-Bionics, Chinese Academy of Sciences, Suzhou 215123, China

[4] Department of NanoEngineering, University of California San Diego, La Jolla, CA 92093, USA

[#] These authors contributed equally to this work: Xiangxi Lou, Penglei Yan, and Binglei Jiao

These authors jointly supervised this work: Panpan Xu, Jinxing Chen

Supplementary Note One

Characterizations

SEM images were obtained with a Zeiss G500 scanning electron microscopy with a n accelerating voltage of 5 kV. TEM images were captured utilizing a FEI Talos F200X transmission electron microscope, employing an acceleration voltage of 200 kV and employing a Ceta 16 M pixel CMOS camera. Powder X-ray diffraction analysis was conducted using a PANalytical X-ray diffractometer at 40 kV and 40 mA, employing Cu Kα radiation (λ = 1.54056 Å). The diffraction patterns were collected in the 2θ range of 5° to 80° with the scanning speed was 2° min^{-1}. Monitoring of the reaction system's temperature was conducted using a JK804 multi-channel temperature tester.

Synchrotron-based XANES and EXAFS spectra were conducted at the SSRF (beamlines 11B). To characterize the valence states and local structural information of Co atoms, the experiment XAS including XANES and EXAFS spectra at Co K-edge spectra were collected in the transmission modes of room temperature. The cobalt K-edge absorption data were all calibrated and aligned using reference Co metal spectra with the maximum value of the first derivative set to 7709 eV. The temperature-dependent XAS experiment was performed using in-house heating cell with an optimized pellet form of sample. To measured XAS spectra, synthesized powders were ground to fine powders and pressed into pellet prior to the experiment.

The quantification of Li and Co content in the samples was achieved using inductively coupled plasma emission spectrometry (PE Optima 8300). To prepare the ICP-OES samples, the sample (10 mg) is first dissolved in 5 ml of concentrated hydrochloric acid. Subsequently, the solution

was diluted in 0.5 wt.% HNO_3 to a concentration of 10 ppm, and then the samples were prepared for ICP-OES measurement.

The ^{1}H and ^{13}C NMR spectra were obtained using a Bruker Advance III HD-400 MHz spectrometer equipped with a BFO smart probe. 11.2 mg of the product and 0.5 mL of d-DMSO were sonicated in an NMR tube until the product dissolved, and 20 μL of dioxane was added as an internal standard. Subsequently, quantitative analysis of the product was performed using ^{1}H NMR. The content of BHET:

$$\frac{\frac{V_{dioxane} \times \rho_{\mathrm{dioxane}}}{\mathrm{M}_{\mathrm{dioxane}}} \times \mathrm{M}_{\mathrm{BHET}} \times \frac{I_{BHET} \times 2}{I_{dioxane}}}{m_{sample}} \quad (1)$$

$$= \frac{\frac{20 \times 10^{-3}\ \mathrm{mL} \times 1.0337\ \mathrm{g\ cm^{-3}}}{88.11\ \mathrm{g\ mol^{-1}}} \times 254.24\ \mathrm{g\ mol^{-1}} \times \frac{2.00 \times 2}{21.41}}{0.0112g} = 0.9951$$

where $V_{dioxane}$ is the volume of dioxane, $\rho_{dioxane}$ is the density of dioxane, $M_{dioxane}$ is the molecular weight of dioxane, M_{BHET} is the molecular weight of BHET, I_{BHET} is the peak area in $\delta = 8.13$ (2H), $I_{dioxane}$ is the peak area in $\delta = 3.56$ (4H).

Thermal catalysis: In the case of thermal catalysis, the reactor was placed in an oil bath to maintain the reaction temperature at 190 °C. All other steps remained consistent with photothermal catalysis. In the UV light system with thermal catalysis, the reaction conditions involved adding an additional UV light with an intensity of 0.01 W cm^{-2} to the thermal catalysis. All other conditions remained consistent with thermal catalysis. The catalytic conditions are listed in Supplementary Table 14.

The PET conversion rate (C_{PET}) and the yield of BHET (Y_{BHET}) was calculated based on following equations.

$$C_{PET} = \frac{\mathrm{W_{PET0}} - W_{PETt}}{\mathrm{W_{PET0}}} \times 100\% \tag{2}$$

$$Y_{BHET} = \frac{W_{BHET}/\mathrm{MW_{BHET}}}{\mathrm{W_{PET0}}/\mathrm{MW_{PET}}} \times 100\% \tag{3}$$

where W_{PET0}, W_{PETt}, and W_{BHET} denote the initial weight of PET, the weight of PET remaining unreacted, and the weight of BHET, respectively. MW_{PET} and MW_{BHET} stand for the molecular weight of the PET repeating unit, 192 g mol^{-1}, and the molecular weight of BHET, 254 g mol^{-1}.

Kinetic studies on the PET glycolysis: The glycolysis of PET can be regarded as a first-order reaction. The reaction equation equations are listed as follows.

$$\mathrm{PET} \overset{k}{\rightarrow} \mathrm{BHET} \tag{4}$$

$$\frac{\mathrm{d}(C_{PET})}{\mathrm{dt}} = -kC_{PET} \tag{5}$$

$$C_{PET} = \mathrm{C_{PET0}e}^{-kt} \tag{6}$$

$$C_{PET} = \mathrm{C_{PET0}}(1 - X) \tag{7}$$

$$\frac{\mathrm{dX}}{\mathrm{dt}} = k(1 - X) \tag{8}$$

$$\ln\frac{1}{1-X} = kt \tag{9}$$

Among them, C_{PET} and C_{PET0} are the concentration of PET at t and 0, respectively; X is the conversion rate of PET; k is the conversion rate of PET at t. Effects of reaction temperature on PET glycolysis with different reaction times are shown in Figs. S10. The slope of the straight line represents the corresponding rate constant. For photothermal catalysis, the rate constants ($k_{photothermal}$) at 170, 175, 180, 185, and 190 ºC are 0.04547, 0.05613, 0.07047, 0.08909 and

0.11458 h^{-1}, respectively. Similarly, the rate constants for the thermal catalysis at 170, 175, 180, 185, and 190 ºC are 0.01853, 0.02643, 0.02974, 0.04025, and 0.04806 h^{-1}, respectively.

The activation energy (Ea) of the reaction can be calculated using the Arrhenius formula.

$$\ln k = \ln \mathrm{A} - \frac{E_a}{\mathrm{R}T} \tag{10}$$

where A, R and T refer to the pre-exponential factor, gas constant (8.314 J $(\mathrm{k \cdot mol})^{-1}$) and reaction temperature in Kelvin, respectively. Activation energies (Ea) were calculated to be 78.7 and 79.4 kJ mol^{-1} from the linear Arrhenius plot.

Cycle performance of catalysts: The experimental procedure followed a standard photothermal catalytic glycolysis protocol. Following the depolymerization step, PET, oligomers, and the catalyst were separated via filtration. The resulting mixture was transferred to a 100 mL beaker, to which 15 mL of N-Methyl-2-pyrrolidone (NMP) was added. The mixture was then heated to 150 °C to ensure complete dissolution of both PET and its oligomers. Subsequently, the catalysts were separated through a hot filtration and washed twice with hot NMP. Throughout the recovery process, there was a marginal loss of catalysts, approximately 15%. To compensate for this loss, fresh catalysts were introduced in the subsequent PET glycolysis cycle.

Long-term stability test: We carried out five long-term parallel experiments, aiming to simulate extended reaction periods. Each experiment-maintained consistency in terms of the number of reactants (5.0 g PET and 10 g EG), catalyst dosage (1 mg $Li_{0.76}CoO_2$), light intensity (0.63 W cm^{-2}), and reaction temperature (170 °C). The sole parameter that varied was the reaction time,

set at 5 hours, 10 hours, 20 hours, 40 hours, and 70 hours, respectively.

Outdoor demonstrations: The experimental conditions were precisely controlled as follows: 50 g of PET, 200 g of EG, and 1 g of $Li_{0.76}CoO_2$ were introduced into a custom-made apparatus. This setup was then exposed to concentrated sunlight for a duration of 20 minutes. The resulting yield of BHET product achieved in this process amounted to 42 g.

Collection and pre-process of real-word plastics: The collected plastics were subjected to a thorough washing process, involving more than three cycles of rinsing with water. Subsequently, the cleaned plastics were dried in a forced-air oven. These plastics were then precision-cut into pieces measuring 0.5 × 0.5 cm each. The specific reaction conditions are summarized in the following Supplementary Table 15.

Economic and Environmental Analysis: The EverBatt model, developed by the Argonne National Laboratory, was employed to conduct a techno-economic and life-cycle assessment of three distinct methods for recycling spent batteries: pyrometallurgy, hydrometallurgy, and direct cathode recycling. This comprehensive model is designed to evaluate the costs and environmental implications of closed-loop battery recycling processes. In addition, our techno-economic analysis has included necessary separation processes. The total cost for LCO separation is calculated to be $1.05 per kilogram.

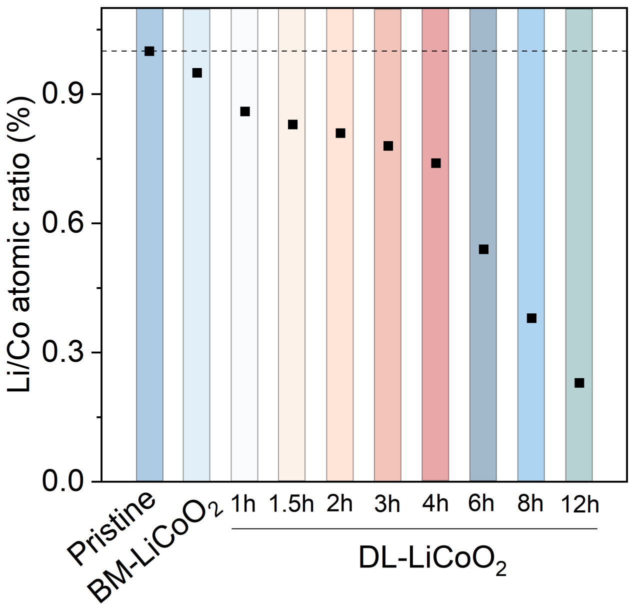

Supplementary Fig. 1 Li/Co (mol/mol) ratio in various samples, including LCO pristine, treated with ball milling for 4 h (BM-$LiCoO_2$), chemical delithiation with 1 h, 1.5 h, 2 h, 3 h, 4 h, 6 h, 8h and 12h.

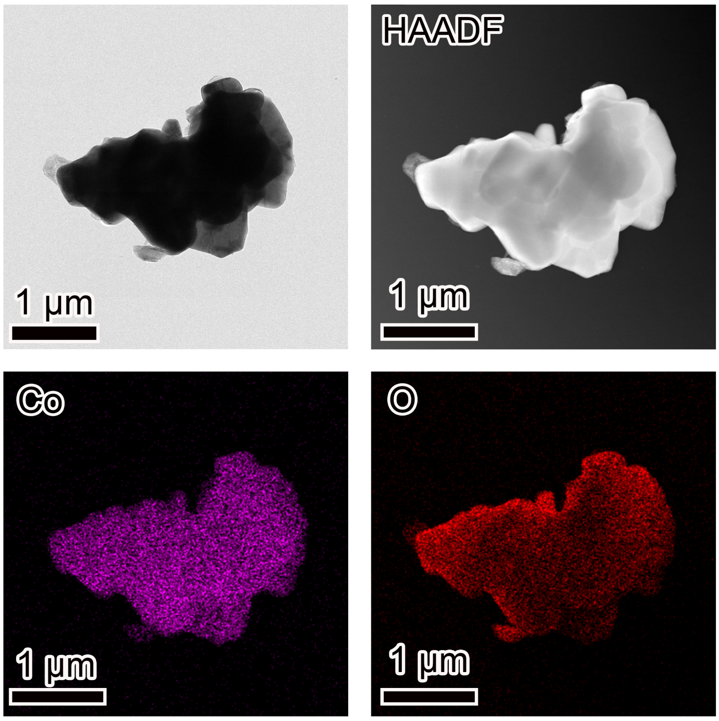

Supplementary Fig. 2 Aberration-corrected HAADF-STEM image and STEM-EDX elemental mapping images for $LiCoO_2$.

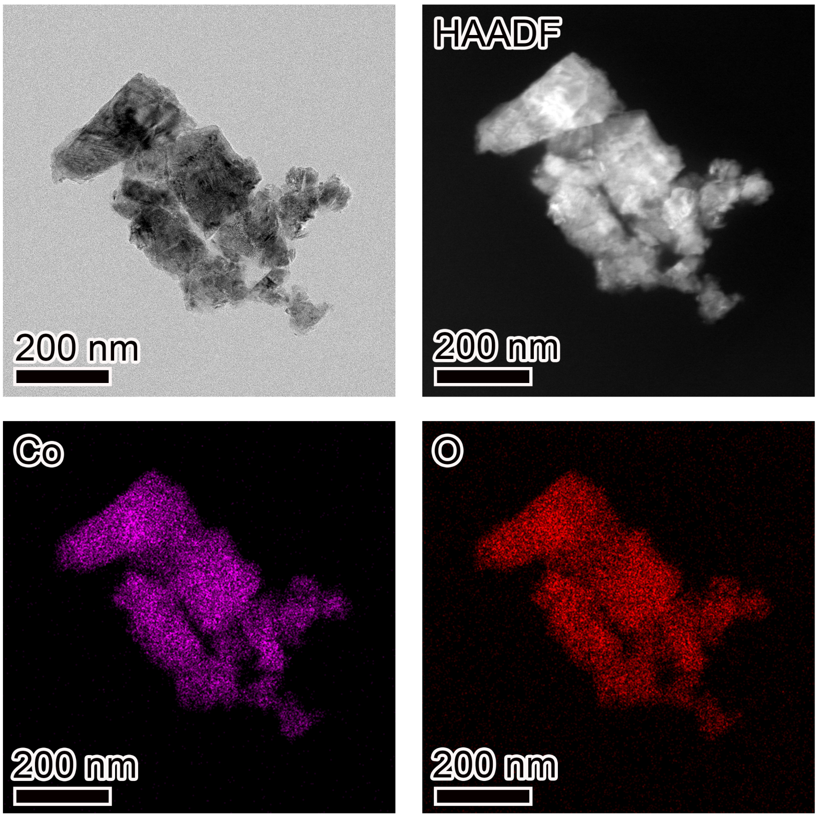

Supplementary Fig. 3 Aberration-corrected HAADF-STEM image and STEM-EDX elemental mapping images for $Li_{0.92}CoO_2$.

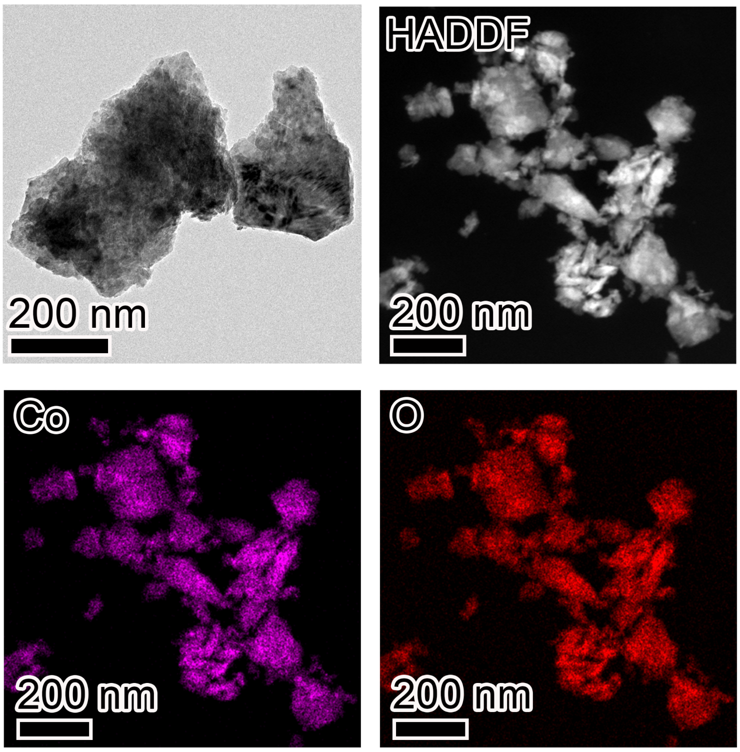

Supplementary Fig. 4 Aberration-corrected HAADF-STEM image and STEM-EDX elemental mapping images for $Li_{0.76}CoO_2$.

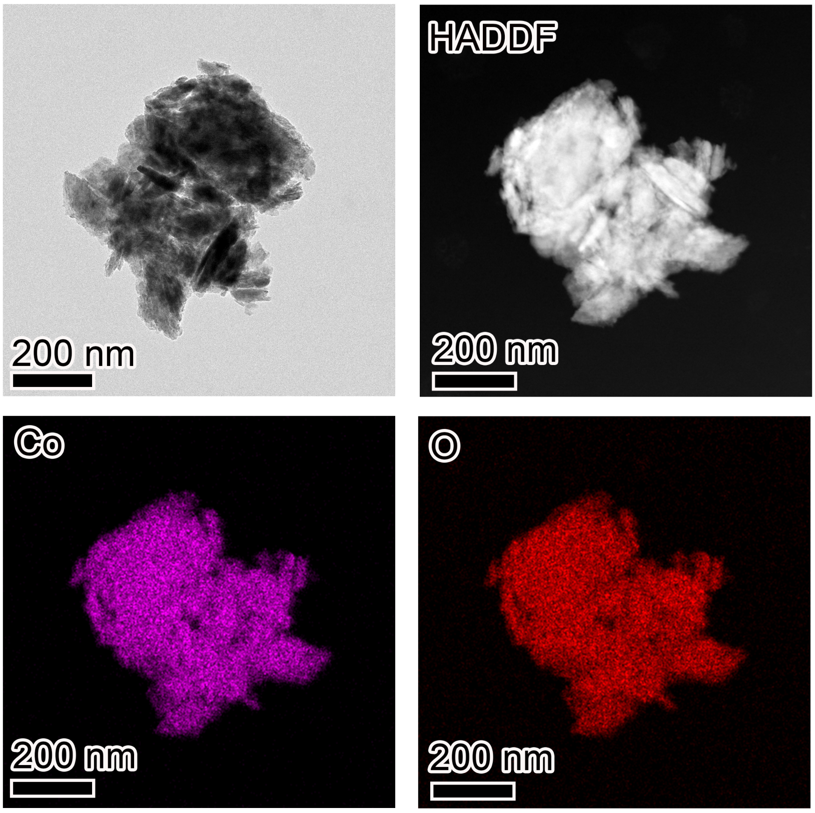

Supplementary Fig. 5 Aberration-corrected HAADF-STEM image and STEM-EDX elemental mapping images for $Li_{0.25}CoO_2$.

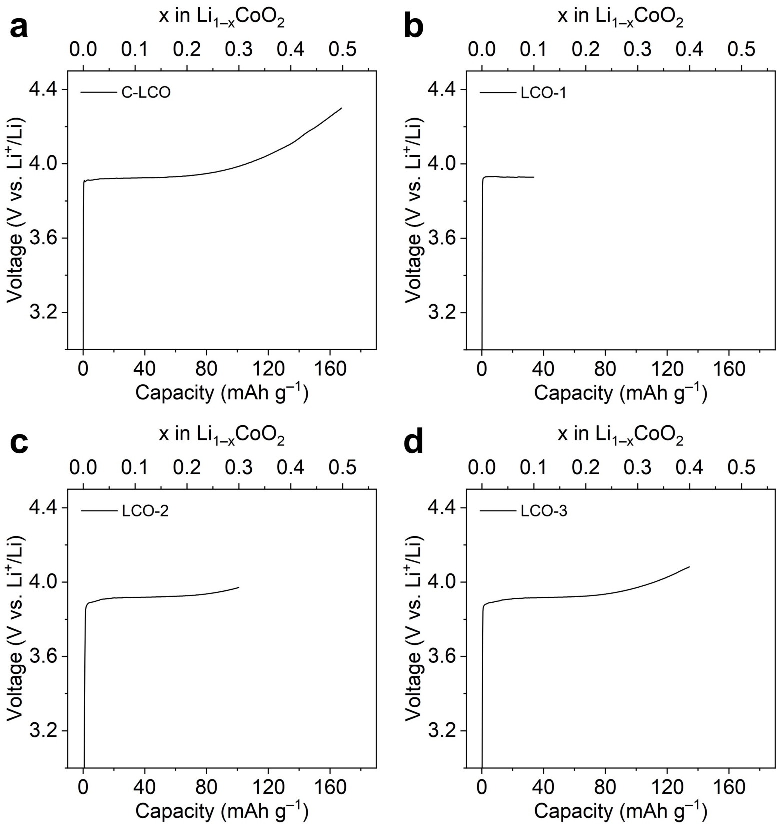

Supplementary Fig. 6 The electrochemical delithiation process. Charge curve at 0.1C with the cutoff voltage of 4.30 V (a), 3.93 V (b), 3.97 V (c) and 4.08 V (d).

Initially, the assembled LCO coin cells were charged at a rate of 0.1C with a cutoff voltage of 4.3 V (Supplementary Fig. 6a). The lithiation degree of the charged LCO cathode was determined to be 0.56 by ICP measurement. The correlation between charging voltage and lithiation degree is illustrated on the upper x-axis in Fig. S6a. To achieve specific lithiation degrees of 0.92, 0.76, and 0.60 for LCO, we set cutoff voltages of 3.93, 3.97 and 4.08 V (Supplementary Figs. 6b–6d). The lithium contents measured in the charged LCO cathodes closely align with the predefined values (Supplementary Table 2).

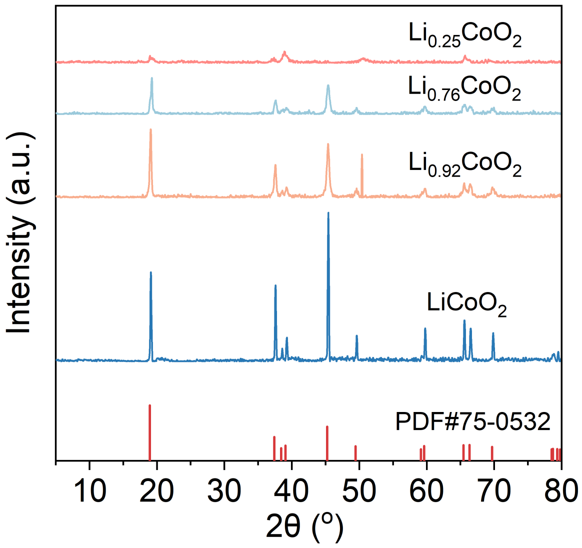

Supplementary Fig. 7 XRD patterns for $LiCoO_2$, $Li_{0.92}CoO_2$, $Li_{0.76}CoO_2$ and $Li_{0.25}CoO_2$.

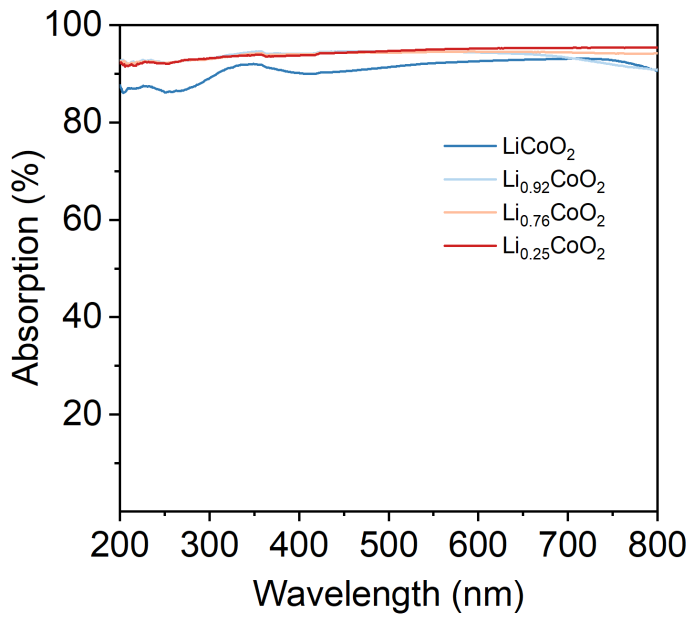

Supplementary Fig. 8 Absorption spectrum of $Li_{1-x}CoO_2$.

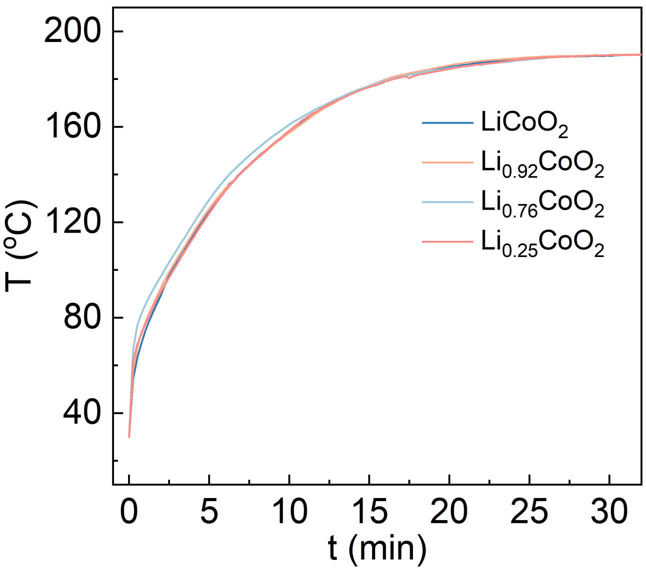

Supplementary Fig. 9 Photo-thermal conversion curve of $Li_{1-x}CoO_2$.

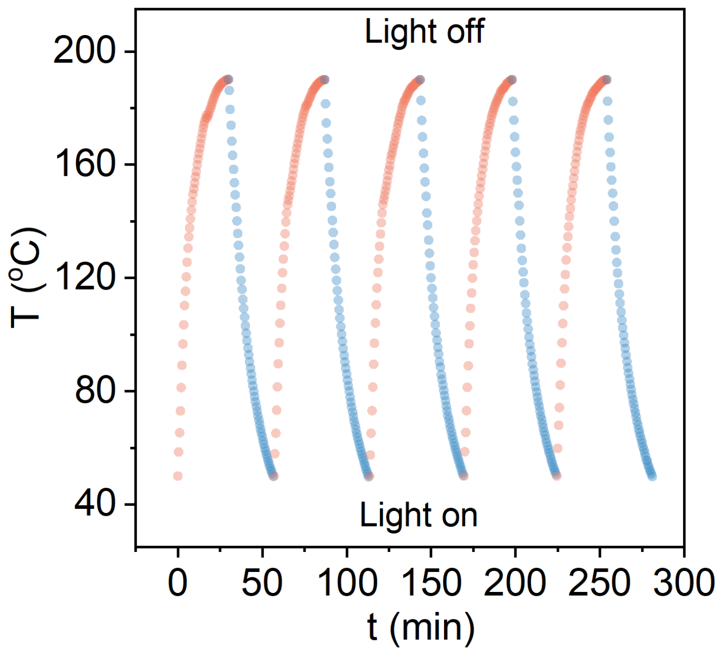

Supplementary Fig. 10 Temperature curve of the EG solution containing 0.4 wt.% of $Li_{0.76}CoO_2$ shined with simulated in five heating-cooling cycles.

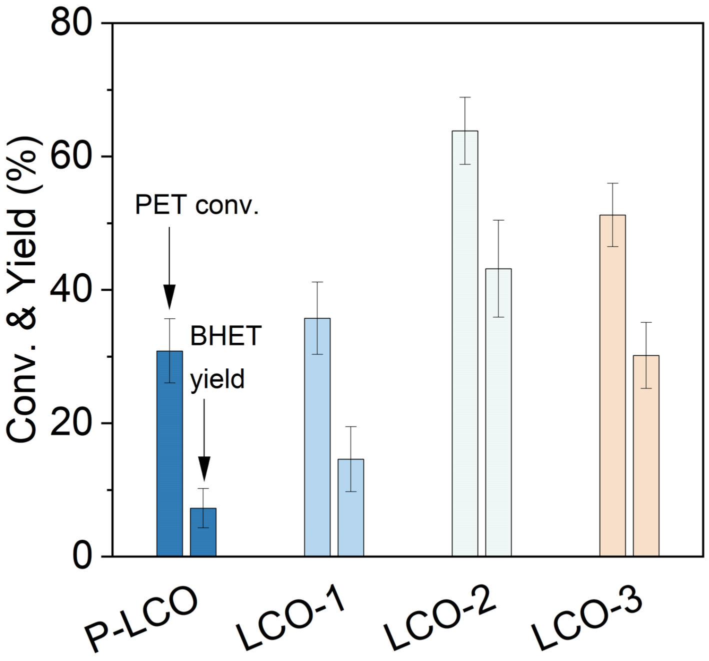

Supplementary Fig. 11 Conversion of PET glycolysis and yield of BHET over LCO-X and pristine LCO catalysts. Commercial PET film was cut into small fragments (0.5 g, 0.5 × 0.5 cm) and immersed in a 2.5 g EG solution with 10 mg of the photothermal catalyst. All error bars in this figure represent the standard deviations of three independent measurements and the bars indicate mean values.

The catalytic activity exhibited an initial increase followed by a decline as the delithiation level increased, mirroring the results obtained from chemical delithiation. Interestingly, the highest catalytic performance was observed at a Li/Co ratio of 0.72. The consistency in catalytic performance rules between catalysts obtained through electrochemical and chemical delithiation reaffirms that the catalyst's performance may be more related to the material's structure than the treatment method employed.

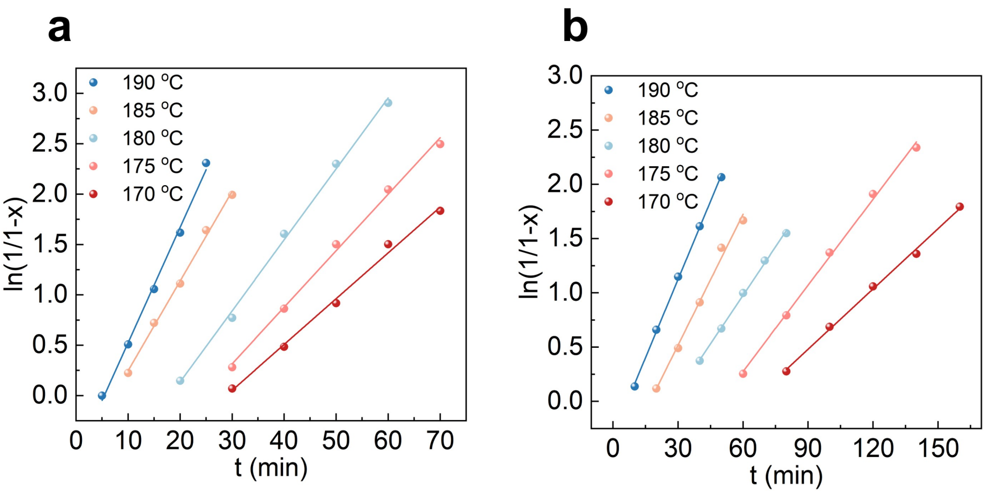

Supplementary Fig. 12 Effect of the temperature on the rate of PET glycolysis. a, Photothermal schemes. **b,** Thermal schemes.

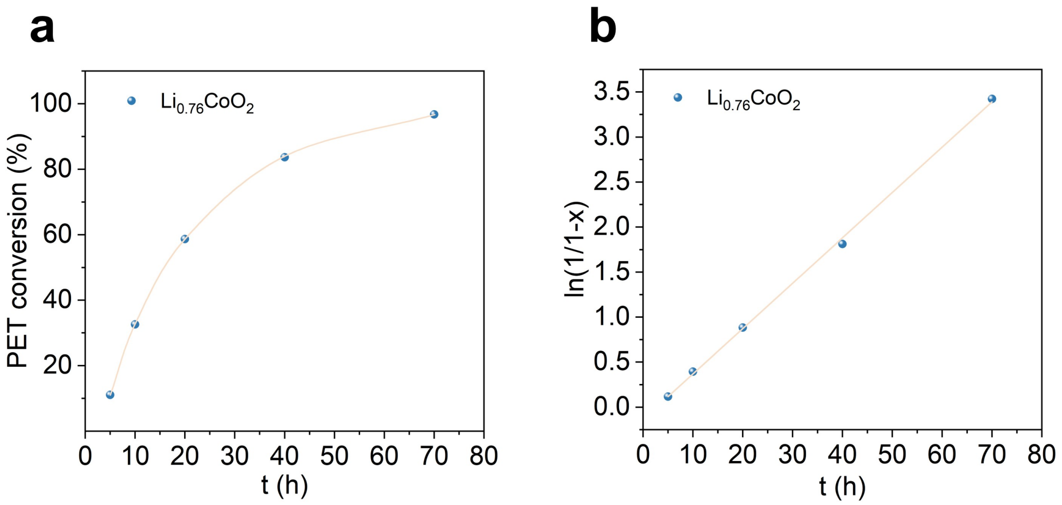

Supplementary Fig. 13 The stability of $Li_{0.76}CoO_2$ with 70 hours of continuous catalytic testing. a, The conversion rates of PET at different times. **b,** The linear fitting of conversion rate versus reaction time.

We recognize the importance of evaluating the long-term stability of the catalyst comprehensively. To address this, we carried out five long-term parallel experiments, aiming to simulate extended reaction periods. Each experiment maintained consistency in terms of the number of reactants (5.0 g PET and 10 g EG), catalyst dosage (1 mg $Li_{0.76}CoO_2$), light intensity (0.63 W cm^{-2}), and reaction temperature (170 °C). The sole parameter that varied was the reaction time, set at 5 hours, 10 hours, 20 hours, 40 hours, and 70 hours, respectively. Notably, to accommodate longer reaction times, we reduced the catalyst dosage to 1 mg while keeping other conditions consistent with the typical reaction conditions.

In Supplementary Fig. 13a, we present the relationship between the conversion rate and reaction time for the five parallel experiments. The gradual increase in the conversion rate of PET with increasing reaction time is evident. Following the literature, PET glycolysis is typically a first-order reaction. The linear fit of the data from Supplementary Fig. 13a demonstrates a perfect linear relationship (Supplementary Fig. 13b), indicating that the catalyst's activity remains robust even under prolonged illumination. This observation assures us of the catalyst's excellent stability over extended reaction times.

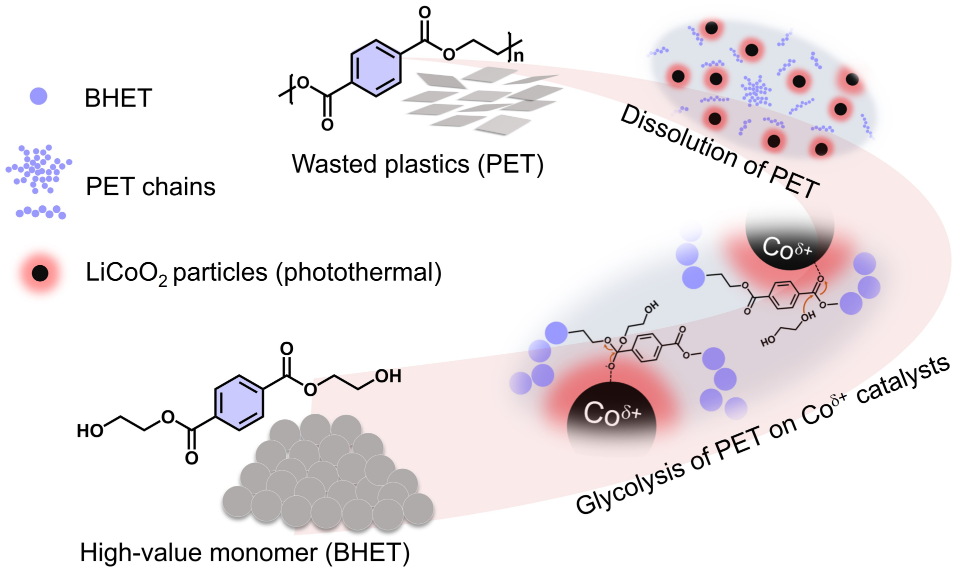

Supplementary Fig. 14 Integrated functionalities of $Li_{0.76}CoO_2$ and the photothermal catalytic mechanism of PET glycolysis over $Li_{0.76}CoO_2$.

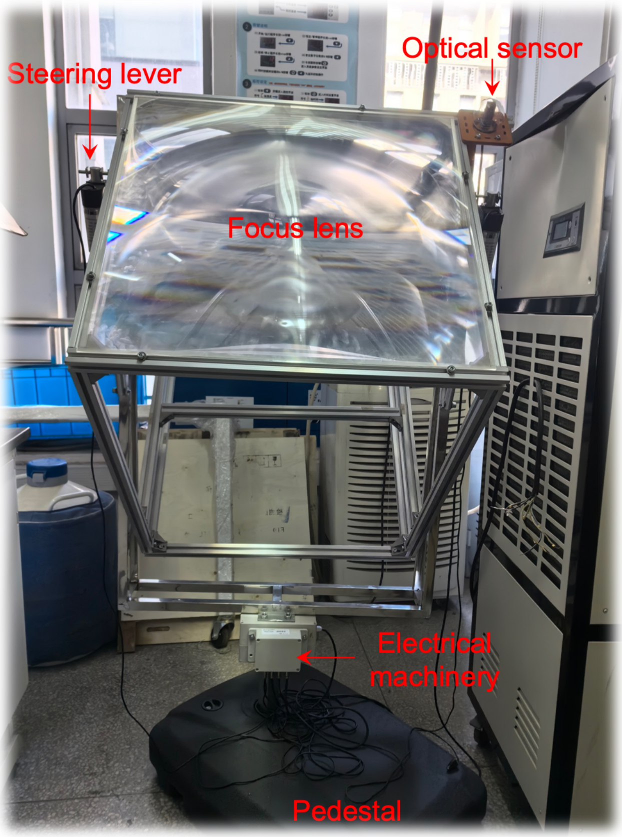

Supplementary Fig. 15 The solar-thermal catalytic system with automatic sun-tracking and adjustable light intensity.

For outdoor experiments, we implemented a solar-thermal catalysis system with solar intensity tracking functionality. This system includes essential components such as a light-tracking sensing and processing system, a light-focusing system, and a servo motor rotation system. Through the adjustment of the azimuth angle of the focusing mirror, the system effectively tracks sunlight, ensuring a stable incident light intensity during outdoor experiments. Currently, the temperature control error of the system is within approximately 7°C, meeting the requirements for the outdoor test.

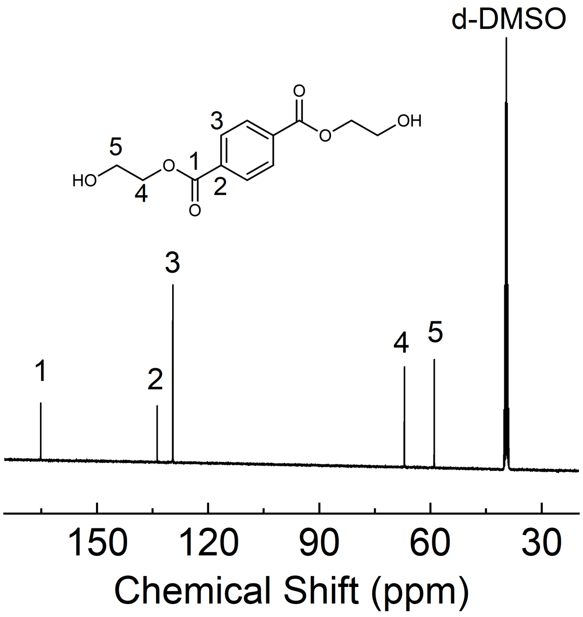

Supplementary Fig. 16 ^{13}C NMR spectra of the product.

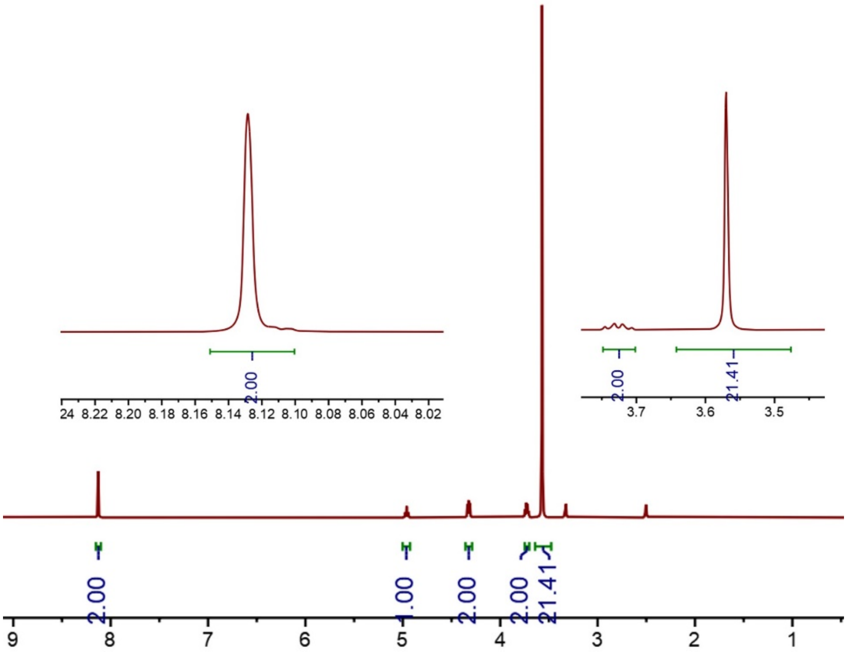

Supplementary Fig. 17 ^{1}H NMR spectra of the BHET and dioxane.

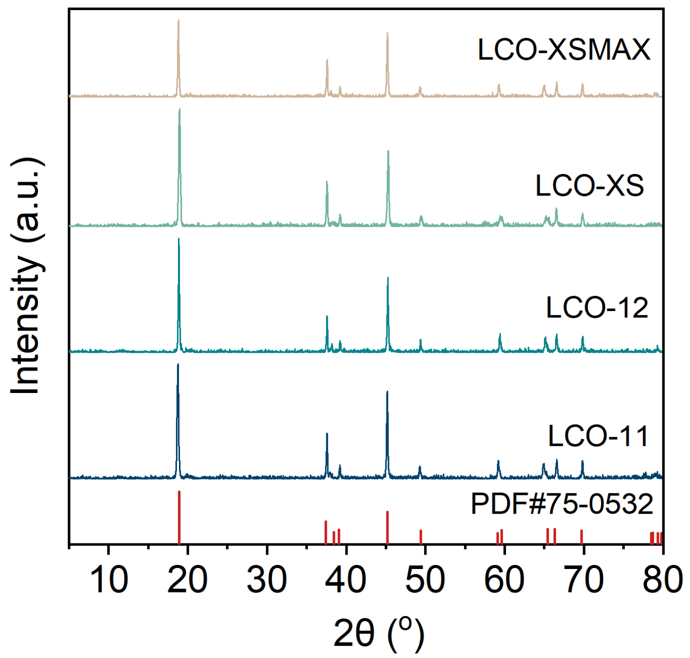

Supplementary Fig. 18 XRD patterns of spent LCO catalysts.

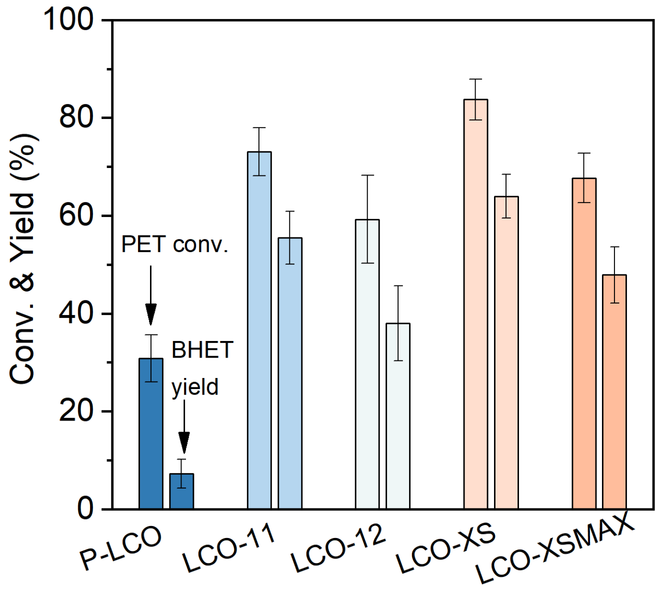

Supplementary Fig. 19 Conversion of PET glycolysis and yield of BHET over spent LCO and pristine LCO catalysts. Commercial PET film was cut into small fragments (0.5 g, 0.5 × 0.5 cm) and immersed in a 2.5 g EG solution with 10 mg of the photothermal catalyst. The solution was then irradiated with a simulated sunlight intensity (0.82 W cm^{-2}) to raise the solution temperature to 190 °C, followed by a 30-minute maintenance period. All error bars in this figure represent the standard deviations of three independent measurements and the bars indicate mean values.

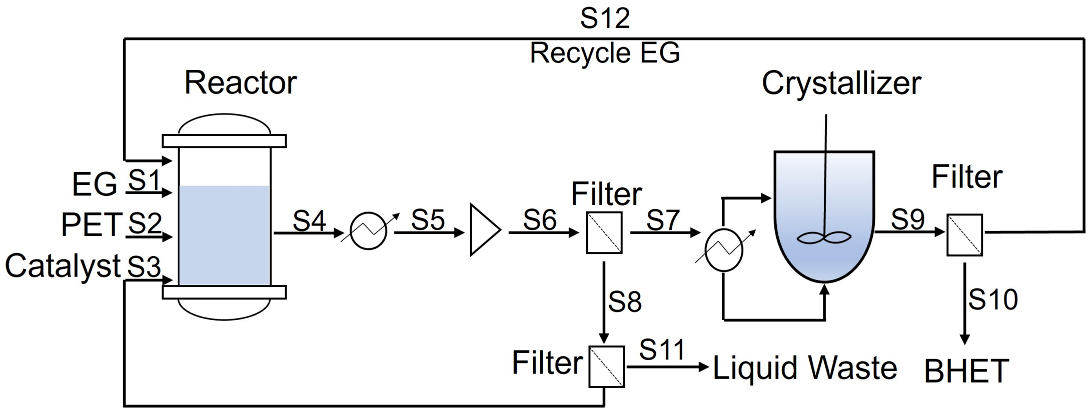

Supplementary Fig. 20 Simplified process flow diagram of the PET glycolysis for a typical photothermal/thermal catalysis.

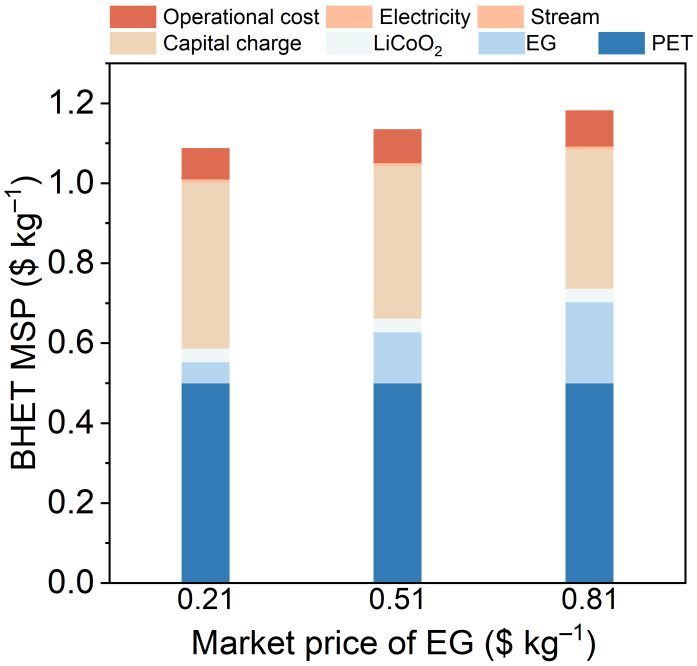

Supplementary Fig. 21 Cost breakdown of the rBHET MSP in the base case process design and as a function of EG price.

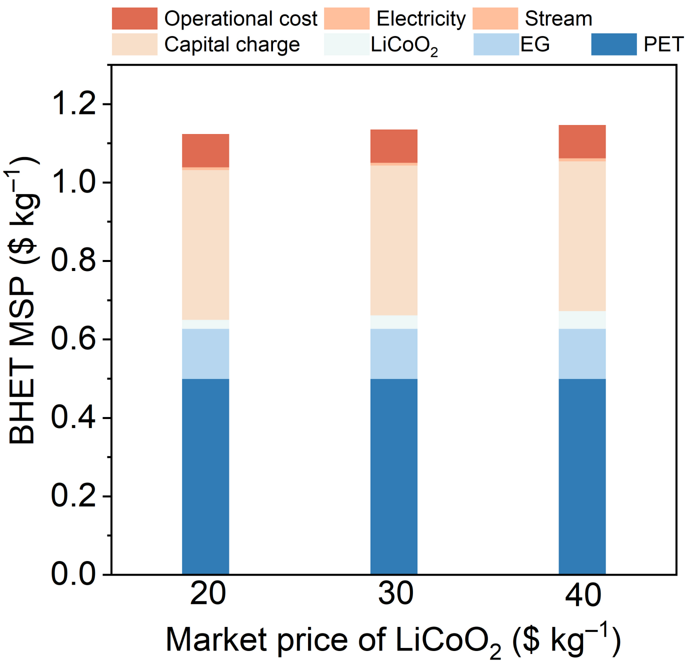

Supplementary Fig. 22 Cost breakdown of the rBHET MSP in the base case process design and as a function of LCO price.

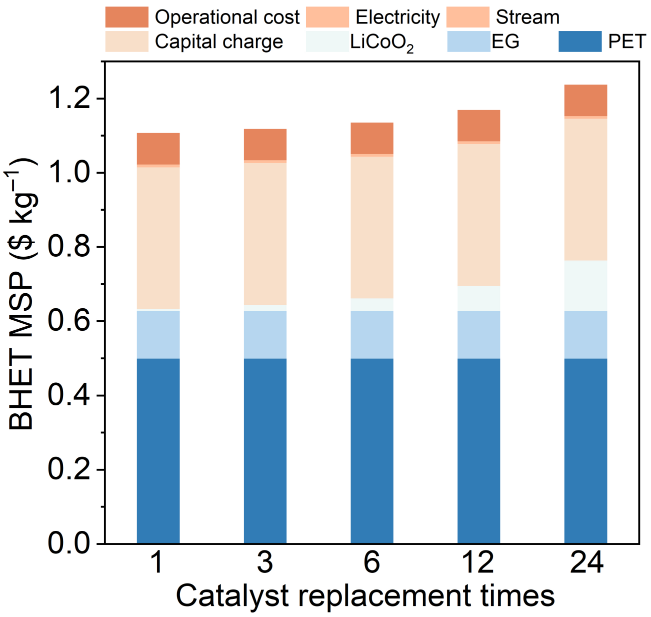

Supplementary Fig. 23 Cost breakdown of the rBHET MSP in the base case process design and as a function of catalyst replacement frequency.

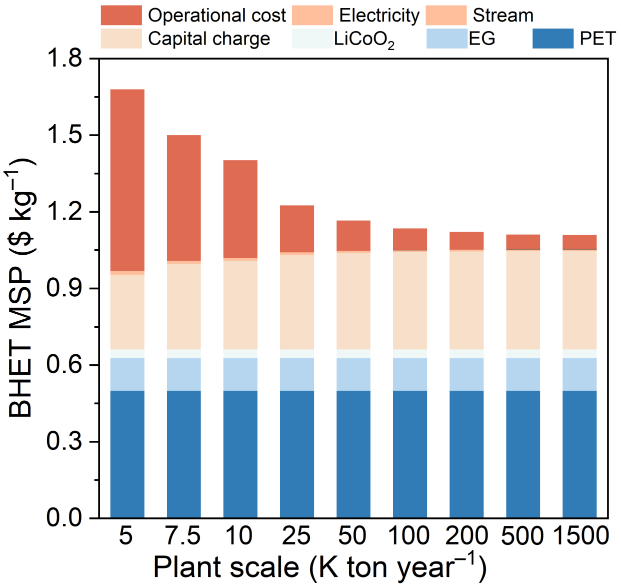

Supplementary Fig. 24 Cost breakdown of the rBHET MSP in the base case process design and as a function of plant size.

Supplementary Table 1 Elemental analysis of $Li_{1-x}CoO_2$.

Sample	Li(wt.%)	Co(wt.%)	Name
$LiCoO_2$	7.36	62.28	$LiCoO_2$
$LiCoO_2$-BM	6.28	58.25	$Li_{0.92}CoO_2$
$LiCoO_2$-BM-4h	5.64	63.29	$Li_{0.76}CoO_2$
$LiCoO_2$-BM-8h	2.60	58.37	$Li_{0.38}CoO_2$
$LiCoO_2$-BM-12h	1.74	59.28	$Li_{0.25}CoO_2$

Supplementary Table 2 Elemental analysis of C-LCO, LCO-1, LCO-2 and LCO-3.

Sample	Li(wt.%)	Co(wt.%)	Li/Co(mol/mol)
C-LCO	3.12	47.34	0.56
LCO-1	4.32	39.49	0.93
LCO-2	4.28	50.45	0.72
LCO-3	3.85	49.99	0.65

Supplementary Table 3 Lattice parameters (a, c) and lattice volume (V) of $LiCoO_2$ and $Li_{0.76}CoO_2$ powders given with reliability factors of weighed (R_{wp}) profiles and goodness of fit (χ^2) values.

	a (Å)	c (Å)	V (Å^3)	R_p (%)	R_{wp} (%)	Gof (χ^2)
$LiCoO_2$	2.8168	14.0536	96.566	1.452	1.34	1.78
$Li_{0.76}CoO_2$	2.8165	14.0650	96.625	1.396	1.29	1.65

Supplementary Table 4 Elemental analysis of LCO-11, LCO-12, LCO-XS and LCO-XSMAX.

Sample	Li(wt.%)	Co(wt.%)	Li/Co(mol/mol)
LCO-11	5.09	64.88	0.67
LCO-12	4.13	41.76	0.84
LCO-XS	4.50	53.01	0.72
LCO-XSMAX	4.75	58.02	0.70

Supplementary Table 5 Detailed PDF of PET recovery process section (base case).

Component	Unit	S1	S2	S3	S4	S5	S6	S7	S8	S9	S10	S11	S12
**Total Stream**	kg/hr	3933.41	11905	23810	118536.91	118536.91	118536.91	94631.2	23905.7	94631.2	15734.47	95.7	78896.73
**Temperature**	°C	25	25	130	190	130	130	130	130	4	4	130	4
**PET**	kg/hr	-	11905	-	-	-	-	-	-	-	-	-	-
**EG**	kg/hr	3933.41	-	-	78976.76	78976.76	78976.76	78896.7	80.02	78896.73	-	80.02	78896.73
**$LiCoO_2$**	kg/hr	-	-	23810	23810	23810	23810	-	23810	-	-	-	-
**H_2O**	kg/hr	-	-	-	-	-	-	-	-	-	-	-	-
**BHET**	kg/hr	-	-	-	15750.15	15750.15	15750.15	15734.47	15.67	-	-	15.67	-
**BHET(S)**	kg/hr	-	-	-	-	-	-	-	-	15734.47	15734.47	-	-

Supplementary Table 6 Yearly operating cost breakdown (base case).

Operating parameters		
Metric/Parameter	Value	Units
Annual operating factor	8000	hrs yr^{-1}
Feedstock contaminants	5%	Wt.%
Feedstock PET	95240	Ton yr^{-1}
Total BHET rate	125876	Ton yr^{-1}
BHET yield	1.32	Ton BHET (ton PET feed) $^{-1}$
PET flake feed mass flow	11905	Kg h^{-1}
BHET production rate	15734.5	Kg h^{-1}

Variable Operating Costs			
Process hierarchy	Raw material/utility	Mass flow (kg h^{-1})	\$ M yr^{-1}
Raw materials			
Feedstock pretreatment	Pet flake feedstock	11905	62.8584
PET depolymerization	EG	3933	16.088
	$LiCoO_2$	23810 ($time^{-1}$)	4.2858
Clarification	Ultrafiltration unit replacement	-	0.266
Crystallization	Membrane replacement	-	0.02
OSBL utilities	HP steam	-	0.862
	Cooling water	-	0.0288
	Grid electricity	-	0.078957
	Subtotal	-	84.488
Total variable operating cost			**84.488**

Fixed operating costs	
Labor & supervision	
Total salaries (managers, supervisors, engineers, technicians, administrative staff)	2.16
Labor burden (90% of total salaries)	1.944
Other overhead	
maintenance	0.136
Property insurance & tax	6.134
Total fixed operating costs	**10.374**
Total operating costs	**94.862**

Supplementary Table 7 Simplified breakdown of the minimum selling price of rBHET in the base case.

**Cost Category**	**Cost Contribution ($ kg rBHET^{-1})**
PET	0.49937
EG	0.12781
$LiCoO_2$	0.03405
Capital charge	0.38180
Stream	0.00685
Electricity	0.00062726
Operational cost	0.08476
**MSP**	**1.135**

Supplementary Table 8 Waste PET sensitivity results.

Case description	Cost category contribution ($ kg rBHET^{-1})							MSP ($ kg rBHET^{-1})
	PET	EG	$LiCoO_2$	Capital charge	Stream	Electricity	Operational cost	
Base case								
Base case	0.49937	0.12781	0.03405	0.3818	0.00685	6.2726E-4	0.08476	1.135
Waste PET sensitivities								
Waste PET cost of 0.22 $ kg^{-1}	0.16646	0.12781	0.03405	0.50637	0.00685	6.2726E-4	0.05858	0.901
Waste PET cost of 1.1 $ kg^{-1}	0.83228	0.12781	0.03405	0.23277	0.00685	6.2726E-4	0.10775	1.342

Supplementary Table 9 EG sensitivity results.

Case description	Cost category contribution ($ kg rBHET^{-1})							MSP ($ kg rBHET^{-1})
	PET	EG	$LiCoO_2$	Capital charge	Stream	Electricity	Operational cost	
Base case								
Base case	0.49937	0.12781	0.03405	0.3818	0.00685	6.2726E-4	0.08476	1.135
EG sensitivities								
EG cost of 0.21 $ kg^{-1}	0.49937	0.05262	0.03405	0.41573	0.00685	6.2726E-4	0.07888	1.088
EG cost of 0.81 $ kg^{-1}	0.49937	0.20298	0.03405	0.34788	0.00685	6.2726E-4	0.09062	1.182

Supplementary Table 10 $LiCoO_2$ sensitivity results.

Case description	Cost category contribution ($ kg rBHET^{-1})							MSP ($ kg rBHET^{-1})
	PET	EG	$LiCoO_2$	Capital charge	Stream	Electricity	Operational cost	
Base case								
Base case	0.49937	0.12781	0.03405	0.3818	0.00685	6.2726E-4	0.08476	1.135
$LiCoO_2$ sensitivities								
$LiCoO_2$ cost of 20 $ kg^{-1}	0.49937	0.12781	0.0227	0.3818	0.00685	6.2726E-4	0.08475	1.124
$LiCoO_2$ cost of 40 $ kg^{-1}	0.49937	0.12781	0.0454	0.3818	0.00685	6.2726E-4	0.08475	1.147

Supplementary Table 11 Number of catalysts sensitivity results.

Case description	Cost category contribution ($ kg rBHET^{-1})							MSP ($ kg rBHET^{-1})
	PET	EG	$LiCoO_2$	Capital charge	Stream	Electricity	Operational cost	
Base case								
Base case	0.49937	0.12781	0.03405	0.38180	0.00685	6.2726E-4	0.08476	1.135
Number of catalysts sensitivities								
Replaced one time per year	0.49937	0.12781	0.00567	0.3818	0.00685	6.2726E-4	0.08476	1.107
Replaced three times per year	0.49937	0.12781	0.01702	0.3818	0.00685	6.2726E-4	0.08476	1.118
Replaced twelve times per year	0.49937	0.12781	0.0681	0.3818	0.00685	6.2726E-4	0.08476	1.169
Replaced twenty-four times per year	0.49937	0.12781	0.13619	0.3818	0.00685	6.2726E-4	0.08476	1.237

Supplementary Table 12 Univariate sensitivity summary.

Sensitivity	Sensitivity parameter range				Bound MSP ($ kg rBHET^{-1})		% MSP Difference (from base case)	
	Units	Lower MSP case	Base case	Higher MSP case	Lower MSP	Higher MSP	Lower %	Upper %
Feedstock cost	$ kg^{-1}	0.22	0.66	1.1	0.901	1.342	-20.66	18.22
Plant size	K ton year^{-1}	1500	100	5	1.109	1.679	-2.29	47.94
EG price	$ kg^{-1}	0.21	0.51	0.81	1.088	1.182	-4.15	4.15
$LiCoO_2$ price	$ kg^{-1}	20	30	40	1.124	1.147	-1.0	0.99
Number of catalysts	time^{-1}	1	6	24	1.107	1.237	-2.5	8.99

Supplementary Table 13 Plant sensitivity results.

Case description	Cost category contribution ($ kg rBHET^{-1})							MSP ($ kg rBHET^{-1})
	PET	EG	$LiCoO_2$	Capital charge	Stream	Electricity	Operational cost	
Base case								
Base case	0.49937	0.12781	0.03405	0.3818	0.00685	6.2726E-4	0.08476	1.135
Plant sensitivities								
Plant size of 5,000 ton per year	0.49938	0.1279	0.03405	0.29235	0.00828	0.00667	0.71084	1.679
Plant size of 7,500 ton per year	0.49937	0.12782	0.03405	0.33567	0.00689	0.00488	0.49189	1.500
Plant size of 10,000 ton per year	0.49937	0.12782	0.03405	0.34637	0.00826	0.00366	0.38221	1.402
Plant size of 25,000 ton per year	0.49938	0.12788	0.03389	0.37014	0.00827	0.00176	0.18434	1.226
Plant size of 50,000 ton per year	0.49937	0.12765	0.03405	0.3784	0.00688	0.00111	0.11901	1.166
Plant size of 200,000 ton per year	0.49938	0.12782	0.03405	0.38341	0.00688	4.9652E-4	0.06991	1.122
Plant size of 500,000 ton per year	0.49937	0.12782	0.03405	0.38538	0.00688	2.78053E-4	0.05738	1.111
Plant size of 1,500,000 ton per year	0.49937	0.12781	0.03405	0.38551	0.00689	7.62657E-5	0.05561	1.109

Supplementary Table 14 The summary of reaction conditions for photothermal and thermal catalysis.

**Fig. 3a**	PET (g)	EG (g)	Catalyst (10 mg)	Temperature (190 °C)	Time (min)
p-$LiCoO_2$	0.5	2.5	p-$LiCoO_2$	0.82 W cm^{-2}	30
$Li_{0.92}CoO_2$	0.5	2.5	$Li_{0.92}CoO_2$	0.82 W cm^{-2}	30
$Li_{0.76}CoO_2$	0.5	2.5	$Li_{0.76}CoO_2$	0.82 W cm^{-2}	30
$Li_{0.38}CoO_2$	0.5	2.5	$Li_{0.38}CoO_2$	0.82 W cm^{-2}	30
$Li_{0.25}CoO_2$	0.5	2.5	$Li_{0.25}CoO_2$	0.82 W cm^{-2}	30
Thermal	0.5	2.5	$Li_{0.76}CoO_2$		10
Thermal + UV	0.5	2.5	$Li_{0.76}CoO_2$	UV (0.01 W cm^{-2})	10
Photothermal	0.5	2.5	$Li_{0.76}CoO_2$	0.82 W cm^{-2}	10

Supplementary Table 15 The summary of reaction conditions for photothermal catalysis of commercial plastics.

Plastic (0.5 g)	EG (g)	Catalyst (10 mg)	Temperature (190 °C)	Time (min)
PET granule	2.5	$Li_{0.76}CoO_2$	0.82 W cm^{-2}	30
PLA	2.5	$Li_{0.76}CoO_2$	0.82 W cm^{-2}	30
PC	2.5	$Li_{0.76}CoO_2$	0.82 W cm^{-2}	30
PET/PE	2.5	$Li_{0.76}CoO_2$	0.82 W cm^{-2}	30
PET/PS	2.5	$Li_{0.76}CoO_2$	0.82 W cm^{-2}	30
PET	2.5	LCO-11	0.82 W cm^{-2}	30
PET	2.5	LCO-12	0.82 W cm^{-2}	30
PET	2.5	LCO-XS	0.82 W cm^{-2}	30
PET	2.5	LCO-XSMAX	0.82 W cm^{-2}	30
Seaside PET bottle	2.5	$Li_{0.76}CoO_2$	0.82 W cm^{-2}	30
Taihu lake PET bottle	2.5	$Li_{0.76}CoO_2$	0.82 W cm^{-2}	30
Dyed PET bottle	2.5	$Li_{0.76}CoO_2$	0.82 W cm^{-2}	30
Dyed PET lunch box	2.5	$Li_{0.76}CoO_2$	0.82 W cm^{-2}	30
Cloth fiber	2.5	$Li_{0.76}CoO_2$	0.82 W cm^{-2}	30
PET packaging film	2.5	$Li_{0.76}CoO_2$	0.82 W cm^{-2}	30
Multi-recycled PET	2.5	$Li_{0.76}CoO_2$	0.82 W cm^{-2}	30
